# Discovery and engineering of bifunctional enzymes for lignocellulose degradation: Metagenomic and computational approaches

**DOI:** 10.1016/j.btre.2025.e00926

**Published:** 2025-09-20

**Authors:** Razieh Goudarzi, Donya Afshar Jahanshahi, Arashk Kavousi, Shohreh Ariaeenejad

**Affiliations:** a Department of Systems and Synthetic Biology, Agricultural Biotechnology Research Institute of Iran (ABRII), Agricultural Research Education and Extension Organization (AREEO), Karaj, Iran; b Laboratory of Complex Biological Systems and Bioinformatics (CBB), Department of Bioinformatics, Institute of Biochemistry and Biophysics (IBB), University of Tehran, Tehran, Iran

**Keywords:** Bifunctional, Promiscuity, Metagenome, Lignocellulose, Biomass hydrolysis

## Abstract

Efficient degradation of lignocellulosic biomass is vital for converting plant-based waste into renewable fuels and chemicals. Owing to its complex composition of cellulose, hemicellulose, and lignin, its enzymatic breakdown often requires multiple enzymes to act synergistically. Bifunctional enzymes that combine two catalytic activities in a single protein offer a promising solution. This review highlights recent advances in the identification and engineering of bifunctional enzymes for lignocellulose degradation, particularly through metagenomics, protein fusion and computational design. Functional pairings, such as cellulase/xylanase, were examined with a focus on their synergistic effects, substrate specificity, and stability. Promiscuous and naturally evolved bifunctional enzymes from extreme or uncultured environments are also discussed. Advances *in silico* modeling and directed evolution have enhanced enzyme properties such as thermostability and substrate range. The review concludes with an outlook on the challenges and opportunities of implementing bifunctional enzymes to improve the economic and technical viability of biomass conversion.

## Highlights

• Bifunctional enzymes improve biomass conversion by integrating two catalytic activities.
• Metagenomics reveals uncultured microbes producing efficient bifunctional enzymes.
• Computational tools accelerate discovery of robust lignocellulolytic bifunctional enzymes.
• The complex lignocellulose structure requires synergistic enzyme action.
• Enzyme promiscuity supports evolution of multifunctional biocatalysts for biomass use.

## Graphical abstract

**Figure:**
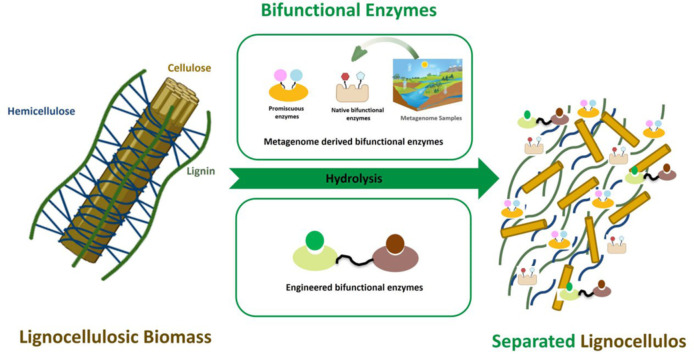

## 1 Introduction

Lignocellulosic biomass (LB), an abundant and renewable natural resource, plays a pivotal role in the eco-friendly production of a wide range of biofuels and biochemicals with minimal or zero carbon and sulfur emissions. Biomass-based industries have also created unique employment opportunities in both developed and developing countries due to the widespread use of biomass in various logistics and industrial operations [1]. LB remains a central focus of international research as a sustainable alternative to fossil-based carbon sources for second-generation biofuels and biochemicals, while simultaneously supporting global food security efforts [2].

In the absence of sufficient carbon-rich renewable energy resources, lignocellulose derived from agricultural residues and forest by-products is an ideal raw material for producing biofuels, chemicals, and biodegradable materials.

However, lignocellulosic waste generated from agro-industries is often resistant to degradation and tends to form complex compounds with other cationic molecules, resulting in environmental pollution [3]. For instance, pulp and paper mills generate between 150 and 200 m³ of effluent, molasses-based distilleries produce 15 liters of effluent per liter of alcohol (totalling 7.5 million tons/year), and agricultural and food industries contribute over 200 billion and 1.3 billion tons of waste per year, respectively [[4], [5], [6]].

This highlights the growing need for the effective biodegradation of agricultural residues. The intricate and compact structure of lignocellulose hinders its hydrolysis. As such, a variety of pretreatment strategies, including biological, physical, chemical, and physicochemical methods, have been employed to enhance its convertibility before further industrial processing [7]. Despite their utility, many of these methods suffer from drawbacks such as high cost, low sugar yields, low efficiency, and the generation of inhibitory by-products [7]. In contrast, enzymatic conversion has gained considerable attention due to its mild operational conditions, minimal equipment requirements, and resistance to inhibitors, offering an efficient approach to biomass valorization [8].

A major limitation of enzymatic saccharification of lignocellulosic feedstocks is their compositional complexity, as they typically consist of 40–45 % cellulose, 25–35 % hemicellulose, and 20–30 % lignin [9]. The strong network of hydrogen bonds and covalent linkages, particularly between hemicellulose and lignin via sugars such as galactose and arabinose, significantly limits enzyme accessibility [10].

Therefore, effective biomass degradation requires a diverse set of enzymes, each targeting a specific structural component. For instance, hemicellulose hydrolysis depends on the concerted action of enzymes such as endo-1,4-β-xylanase (EC 3.2.1.8) and exo-1,4-β-xylosidase (EC 3.2.1.37) [11], while cellulose breakdown is facilitated by endoglucanases (EC 3.2.1.4), exoglucanases (EC 3.2.1.91), and β-glucosidase (EC 3.2.1.21) [10]. Lignin, the most recalcitrant component, is degraded mainly by oxidative enzymes including laccases, manganese peroxidases (MnP), lignin peroxidases (LiP), and versatile peroxidases (VP), predominantly secreted by white-rot fungi [12,13].

Given this complexity, synergistic enzyme activity is essential for efficient hydrolysis of biomass. Strategies such as enzyme cocktails, fusion proteins, and multifunctional and bifunctional enzymes have emerged as promising solutions.

Recent advances have also demonstrated integrating novel enzymes, particularly metagenome-derived cellulases and bifunctional xylanase/β-glucosidase, with advanced immobilization strategies, such as graphene oxide or nanocellulose-based hydrogels, can significantly improve enzymatic hydrolysis efficiency and, in some cases, enhance downstream processes, such as ethanol production [[14], [15], [16]] Recent studies have also shown that immobilizing xylanases on advanced bio-based or nanostructured supports, including polyvinyl alcohol gum Arabic hydrogel nanocomposites, organically modified mesoporous silica nanoparticles, and bio-based hydrogels, can greatly enhance enzyme stability, catalytic efficiency, and application performance in diverse sectors ranging from lignocellulosic biomass hydrolysis to poultry feed improvement [[17], [18], [19]].

Reports indicate that exoglucanases and endoglucanases cooperate to convert cellulose into cellobiose and ultimately glucose through the action of β-glucosidase. The prevalence of xylans in grass, hardwood, and softwood biomass underscores the importance of incorporating CAZymes with broad substrate specificity into enzyme mixtures, allowing for effective degradation across diverse feedstocks [20]. Cooperative action between cellulases and hemicellulases, particularly xylanases, further enhances cellulose accessibility by disrupting the hemicellulose barriers [21,22].

**Fig. 1:**
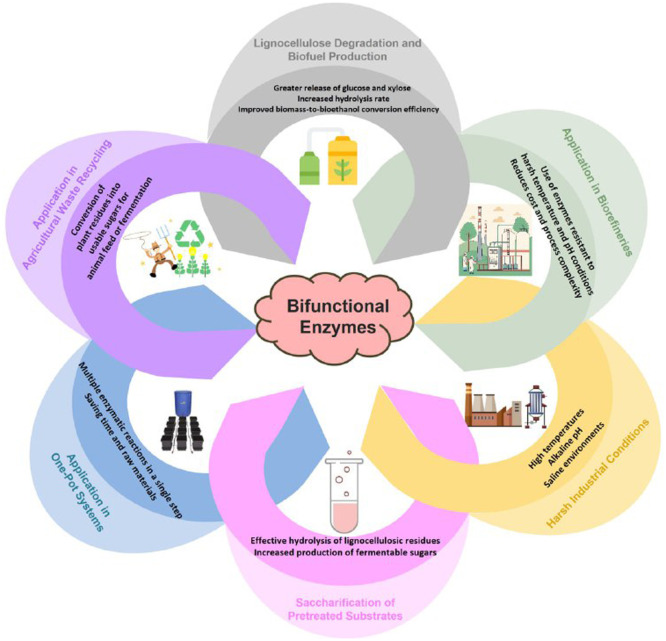
Schematic overview of the diverse industrial and environmental applications of bifunctional enzymes in lignocellulosic biomass valorization. These enzymes contribute to enhanced hydrolysis rates, improved fermentable sugar yields, and increased process efficiency under various conditions such as harsh industrial environments, biorefineries, agricultural waste recycling, and one-pot systems.

The widespread applications of bifunctional enzymes in enhancing biomass hydrolysis, biofuel production, agricultural waste recycling, and tolerance to harsh industrial conditions are summarized in Fig. 1.

Bifunctional and multifunctional enzymes have gained significant interest in industrial bioconversion processes due to their capacity to catalyze multiple reactions, thereby reducing the number of enzymes needed. These enzymes may naturally possess multiple functions (moonlighting or promiscuous enzymes) or be artificially constructed through gene fusion [23].

Moonlighting enzymes contribute to non-catalytic roles, such as structural or regulatory functions, whereas promiscuous enzymes catalyze multiple reactions using the same active site. Several promising bifunctional enzymes have been identified. For instance, a thermostable cellobiohydrolase/xylanase was isolated and characterized for efficient wheat straw hydrolysis [24], and an acetyl xylan esterase/arabinofuranosidase was shown to significantly enhance lignocellulose breakdown [25]. Through protein engineering, fusion enzymes combining two or more activities have been created to synergistically degrade biomass components [[26], [27], [28]].

While many bifunctional enzymes have been discovered through conventional screening of culturable microorganisms, a vast array of functional genes remains hidden in uncultivated microbial communities [29,30]. Therefore, the identification of novel enzymes with industrial potential has become a research priority. Metagenomics offers a powerful, culture-independent approach to explore microbial diversity and access hidden biocatalytic potential [31,32]. Several bifunctional enzymes discovered through metagenomic screening have been patented and commercialized, underscoring the value of this approach [33]. Previous studies have found that bifunctional lignocellulolytic enzymes can achieve cost-effective and highly efficient hydrolysis of lignocellulosic substrates for industrial applications [[34], [35], [36]]. Furthermore, in the era of big data, *in silico* methods and machine learning are facilitating the discovery of enzymes with high catalytic efficiency and robustness, ultimately reducing the reliance on costly enzyme cocktails[37,38]. Recent studies have demonstrated the potential of machine-learning-assisted computational pipelines for targeted enzyme discovery in metagenomic datasets. For example, a generalized machine‐learning–aided framework has been developed for the targeted identification of industrial enzymes, such as xylanases, with temperature‐dependent activity from metagenomic data. Similarly, a computational approach based on pseudo amino acid composition features has been proposed to accurately predict xylanase activity in *Bacillus subtilis* strains, underscoring the growing role of AI-driven tools in enzyme functional prediction and screening [39,40].

Given the challenges of degrading recalcitrant lignocellulosic biomass, there is growing interest in multifunctional and bifunctional enzymes and the technologies used for their identification. This review critically discusses the current advances in bifunctional enzyme discovery, their biochemical properties, and their applications in biorefineries operating under harsh industrial conditions.

## 2 Bifunctional enzymes for the lignocellulose-based applications

Enzymes often evolve to possess bifunctional or multifunctional capabilities to enhance metabolic efficiency. Bifunctional enzymes possess two distinct catalytic capacities and catalyze two consecutive reactions. Therefore, these enzymes exhibit unique properties that are useful in industrial applications [41]. As a result of evolution, bifunctional and multifunctional enzymes have appeared over time as a combination of genes encoding enzymes that catalyze sequential reactions [41]. Generally, bifunctional enzymes demonstrate synergistic effects in the degradation of substrates, and their assemblies control the reaction and prevent metabolic crosstalk between competing pathways [42]. Enzymes can be categorized into different groups based on their structures and functions.

### 2.1 Native bifunctional enzymes

Natural environments are a major source of enzymes with numerous applications. Traditional methods depend on establishing pure cultures, which limits access to microbial diversity. It has been shown that nearly 99 % of microbes have never been cultured in the laboratory [43]. Therefore, millions of microbial species remain to be studied. The culture-dependent method is based on the isolation of the strain from nature, followed by its growth under the desired conditions and detection of the desired organism [44]. Due to the limitations of cultivation methods, such as high cost, time consumption, and limited access to the entire community of microorganisms, they are currently biased.

To date, multiple bifunctional enzymes have been identified using this approach (Table 1). Rattu et al. reported a thermostable bifunctional gene encoding both CMCase and xylanase, derived from *Bacillus halodurans* TSLV1. This enzyme, which belongs to the GH5 family, functions as an endo-acting catalyst with exoglucanase activity and features a single catalytic domain alongside a carbohydrate-binding module [45]. It can convert corn cobs and wheat bran substrates and produce fermentable sugars, highlighting the enzyme capability for bioethanol and biochemical production [45]. In another study, the cellulase/xylanase enzyme CtCel5E was shown to be a bifunctional enzyme generated by *Clostridium thermocellum*. The highest cellulase/xylanase activity was observed at pH 5 and 6. Biofuel production and biomass degradation may be facilitated by these multifunctional enzymes. The catalytic domain of CtCel5E-T1 was amplified using primers P3 and P4. With P5/P6 and P7/P8, the catalytic domains of CtCel5E- and CtCel5E-T3 were amplified along with either the C- or N-terminal peptide linker. The PCR products were cloned into pHTPP13 using EcoRI and XhoI sites. DNA sequencing was used to identify the correct colonies from the pHTPP13 constructs that were transformed into *Escherichia coli* DH5α [46]. In a recent study, Han et al. identified a novel bifunctional cellobiohydrolase-xylanase from the GH7 family of glycoside hydrolases from *C. thermophilum*, which exhibited high thermostability [24]. The enzyme generated oligosaccharides during the continuous saccharification of lignocellulosic substrates making the enzyme a good candidate for biofuels and bio-based chemicals [24]. Shi et al. reported the production of a new bifunctional xylanase/glucanase from *Paenibacillus* sp. A homology modeling approach was used to construct a three-dimensional model of the enzyme, which was refined using MD simulations. These results support the view that xylanase is a bifunctional xylanase/glucanase with a single catalytic domain, in contrast to most bifunctional/multifunctional enzymes that contain several distinct catalytic domains. In recombinant XynBE18, the optimal pH for xylanase activity ranged from 7.5 to 9.0, whereas the optimal pH for glucanase activity was 6.5 [47]. Additionally, CtCel7 is a novel hyperthermostable bifunctional cellobiohydrolase-xylanase enzyme is a recombinant enzyme that hydrolyzes cellulosic feedstocks into fermentable sugars, which are important for the production of food, biofuels, and biochemicals. CtCel7 was added to a commercial cellulase cocktail to improve the hydrolysis of the pretreated wheat straw. This enzyme can be used as a biocatalyst for biomass conversion in practical biotechnological applications. Based on these results, CtCel7 is a promising candidate for the biotechnological production of biofuels and bio-based chemicals [24]. In another report, the ruminant xylanase gene XynA-7 was co-expressed with the bifunctional cellulase gene CelA-5 in ruminant fungi. Cellulase and xylanase were amplified from *Neocallimastix* sp. and the cellulase activity performed best at pH 6.0 and 40 °C, and xylanase activity showed the highest activity at pH 6.5 and 50 °C. This enzyme can degrade CMC, xylan, and lichen [48]. In a previous study, a novel acetyl xylan esterase/arabinofuranosidase with carbohydrate esterase domains and glycosyl hydrolase family 62 domains was produced from *Penicillium chrysogenum* P33 [25]. According to sequence alignment, in addition to esterase activity, GH62 contains a GH62 domain for α-l-arabinofuranosidase activity, with optimum pH and temperature values of 7.0 and 50 °C, respectively. Compared to commercial cellulase, the enzyme produced 51 % more glucose from delignified corn stover, indicating superior hydrolysis [25]. A bifunctional enzyme with processive endoglucanase and xylanase activities and two carbohydrate-binding modules was isolated from *H. chejuensis.* The enzyme generated cellobiose and xylooligosaccharide degradation from cellulosic substrates and glucuronoxylans, respectively [49]. As reported by Pe´rez-Avalos et al. a bifunctional cellulase/xylanase secreted from *Cellulomonas flavigena* CDBB-531 displayed a high similarity with the GH 10 family and had an optimal temperature of 50 °C for xylanase activity and pH optima of 6 and 9 for cellulase and xylanase activities, respectively [50]. In a previous study, Xue et al. introduced a novel Xylanase/ β -Glucanase produced from *Caldicellulosiruptor bescii*. This bifunctional novel enzyme is used for the hydrolysis of multiple substrates with different glycosidic linkages [51]. Liu et al. reported a novel GH43 family enzyme with both β-xylosidase and α-L-arabinofuranosidase activities from *Bacillus pumilus.* The enzyme was cold-active, showed strong tolerance to xylose, and demonstrated a high synergistic effect with endo-xylanase to hydrolyze beechwood xylan [52]. As described in previous studies, *Humicola grisea* var.*thermoidea* was used to produce a novel bifunctional enzyme with -xylosidase and -arabinfuranosidase activities. The purified enzyme was thermostable and exhibited strong tolerance to xylose [51]. In another study, a bifunctional β-xylosidase/α-l-arabinofuranosidase was isolated from *Colletotrichum graminicola*, which exhibited high stability towards NaCl and maintained high activity over a wide pH range. This enzyme hydrolyzes xylooligosaccharides containing up to six pentoses [53]. Belfaquih et al. reported the production of a bifunctional -xylosidase/xylose isomerase from *Streptomyces sp* which acts as an exoenzyme on xylooligosaccharides [54]. Sermsathanaswadi et al. reported the isolation of a new xylanase/β-glucanase from *Paenibacillus curdlanolyticus*, which could bind soluble and insoluble polysaccharides. This contributes to the high catalytic efficiency and substrate-binding ability of the enzyme, indicating its capability to convert lignocellulose and generate fermentable sugars [55]. A thermostable xylanase/cellulase enzyme from the GH10 family enhances the saccharification efficiency of lignocellulose-degrading enzymes. The enzyme exhibited high catalytic performance in the presence of various lignocellulose substrates, such as beechwood xylan, wheat arabinoxylan, filter paper, and barley β-glucan, and efficiently degraded pretreated corn stover and sugarcane bagasse [56]. The hyperthermophilic bacterium *Dictyoglomus turgidum* was used to identify a novel endomannanase/ endoglucanase. The purified recombinant enzyme was highly thermostable and resistant to inhibitors; therefore, it was introduced as a potential enzyme for the conversion of lignocellulosic biomass into fermentable sugars and for biofuel production [57].

**Table 1:** Some novel identified multi and bifunctional enzymes with application in lignocellulose industries.

Bifunctional enzyme	Source	Application in lignocellulos industry	Performance metrics or benefits	Ref
Cellulase/Xylanase	*Bacillus halodurans* TSLV1	Hydrolysis of corn cobs and wheat bran	Purification yield with 2-fold purity increase	[45]
Cellulase/Xylanase	*Clostridium thermocellum*	Biomass conversion	Increase in cellulose activity	[46]
Cellobiohydrolase/ Xylanase	*Chaetomium thermophilum*	Saccharification of pretreated wheat straw	Marginal increase in hydrolysis & reduce sugar yield	[24]
Xylanase/Glucanase	*Paenibacillus sp*	Hydrolysis of cellulosic substrates	Reduce sugar, High purity (10.6-fold), final yield of 9.2 % & specific activity 59.6 U/mg	[47]
Cellulase/Xylanase	*Neocallimastix* sp. GMLF7	Hydrolysis of cellulosic substrates	Reduce sugar, improving the efficiency of feed utilization and animal performance	[48]
Acetyl xylan esterase/Arabinofuranosidase	*Penicillium chrysogenum*	Saccharification of cellulose and xylan	Stable (pH 6–9), metal-tolerant, improves glucose yield via synergy with xylanase	[25]
Endoglucanase and Xylanase	*Hahella chejuensis* KCTC 2396	Hydrolysis of cellulose and hemicellulose	More efficient hydrolysis and increase in enzyme activity	[49]
Endoglucanase/Endoxylanase	*Cellulomonas flavigena*	Hydrolysis of cellulose and hemicellulose	Purified 39-fold with 11 % final activity yield	[50]
Xylanase/ β -Glucanase	*Caldicellulosiruptor bescii*	Hydrolysis of cellulose and hemicellulose	Viscosity reduction (16–24 %)	[51]
β-xylosidase/α-L arabinofuranosidase	*Bacillus pumilus*	Saccharification of xylan at low temperature.	Synergistic reaction yielded more xylose; highest synergy degree (16.21)	[52]
β -xylosidase/α-arabinfuranosidase	*Humicola grisea* var.*thermoidea*	Sugarcane bagasse hydrolysis	Increase in hydrolysis, commercial cellulase, thermostable & xylose-tolerant (29 %)	[59]
β-xylosidase/α-l arabinofuranosidase	*Colletotrichum graminicola*	Hydrolysis of xylooligosaccharides	Stable up to 70 °C, salt-tolerant	[53]
β -xylosidase/Xylose isomerase	*Streptomyces sp*. EC 10	Hydrolysis of cellulose and hemicellulose	The yield ranges from 20 % to 100 % under different conditions	[54]
Xylanase/ β-Glucanase	*Paenibacillus curdlanolyticus B-6*	Degradation of untreated corn stover and sugarcane bagasse	Reduce sugar, high specific activity on soluble Xylan (1487 U/μmol)	[55]
Cellulase/Xylanase	*Bacillus* sp. KW1	Degradation of pretreated corn stover and sugar cane bagasse	The enzyme mutant showed higher activity & sugar yield from biomass	[56]
Endomannanase/ Endoglucanase	*Dictyoglomus turgidum*	Hydrolysis of cellulose and hemicellulose	High thermal & pH stability; optimal activity at 70 °C & pH 5.4	[57]
Cellulase/Mannanase/Xylanase	*Paenibacillus curdlanolyticus*	Hydrolysis of untreated corn hull and rice straw	Oligosaccharides as value-added products	[58]
Xylobiohydrolase/Glucuronoxylanase	*T. thermophila ATCC 42,464*	Lignocellulose degradation	Enhance xylanase activity, synergistic action improves xylo-oligomer, arabinose release, supports biomass valorization	[60]
Cellulose/Xylanase Exogluclanase	*Thielavia terrestris*	Enzymatic Hydrolysis of Agroindustrial Derivatives	Active on complex substrates; exo/endo activity, suitable for industrial use	[61]
Cellulolytic/Hemicellulolytic	*Talaromyces rugulosus.*	Cellulose hydrolysis	Boosts cellulose hydrolysis, potential booster in enzyme cocktails	[62]
α-l-arabinofuranosidase/Xylanase/β-glucanase	*Caldicellulosiruptor bescii*	Hydrolyzing unmodified wheat bran and corn cob	Thermostable, synergistic hydrolysis of xylan-rich residues without pretreatment, efficient sugar release	[63]
β-xylosidase/Endoxylanase	*thermophilic Geobacillus* sp. *WSUCF1*	Lignocellulosic biomass conversion.	Thermostable bifunctional enzyme, 77 % xylan conversion, suitable for value-added bioproducts	[64]
Cellulose/Xylan	*Trichoderma reesei*	Biomass enzymatic saccharification and bioethanol conversion	Simultaneous cellulase/xylanase activity, enhanced saccharification, suitable for bioethanol production	[65]
Endoglucanase/Bifunctional cellulase	*microbial-derived*	Synergistic activity on wheat straw	Bf2006 showed 3-in-1 activity: endo (130.78), exo (1406.36), β-glu (1119.25) U/mg and potential for industrial lignocellulose conversion	[66]
CE15 glucuronoyl esterases /Xylanases	*Artolenzites elegans and Trametes ljubarskyi*	Lignocellulose saccharification	Enhance saccharification by 57–61 μM, synergy raised xylose yield (10 % vs 6 %), increase +55 % xylotriose	[67]

In addition, searching for industrially useful multifunctional enzymes with the ability to tolerate extreme pH and temperature is crucial to overcome obstacles in biomass conversion, such as the hydrolysis of a variety of substrates that may exist in the biomass. Phakeenuya et al. reported a novel multifunctional enzyme with endo/exo cellulase, mannanase, and xylanase from *Paenibacillus curdlanolyticus*. The new enzyme was subjected to hydrolysis of various substrates and generated cellobiose, mannotriose, and xylotriose. In addition, after hydrolysis of untreated corn hulls and rice straw, the main products were xylo- and cello-oligosaccharides [58].

### 2.2 Fusion approach for creating bifunctional enzymes

Many studies have used genetic fusion approaches to construct bifunctional and multifunctional enzymes. This method involves the combination of two or more protein domains in one cell, and the resulting biocatalyst can catalyze a cascade of reactions [68]. This technique has advantages over the use of a mixture of individual monofunctional enzymes and can facilitate multiple reactions in the biodegradation industry. This method is feasible for reducing the number of enzymes required for feedstock biodegradation [69]. The fusion of distinct catalytic functions within a single protein, regulated by a single promoter, is a common strategy, particularly when the synchronized expression of these activities is beneficial.

There are two methods for gene fusion according to molecular biology techniques. In insertional fusion, one domain is connected to the middle of the other domain using one or two linkers to achieve chimerization [69]. This leads to formation of stable, rigid structures that are resistant to denaturation. Finding the “cut” site for insertion should be selected properly; otherwise, domain folding would disrupt, which makes this technique difficult to design [69]. Another technique combines enzymes using end-to-end genetic fusion. Wherein, the N- or C-terminal regions of the enzymes are joined together and act as “bridges”. This method is advantageous because of its ease of processing and flexibility. Linker peptides are preferred for acquiring maximal flexibility by adopting an extended conformation [28].

Several bifunctional enzymes have been constructed to convert lignocellulose and increase sugar release from these substrates (Table 2). In another study, endoglucanase, exoglucanase, and different carbohydrate-binding modules were fused to design several bifunctional cellulases. The fused enzymes exhibited high potential for the degradation of lignocellulosic substrates, such as rice straw and wheat straw [26]. Cell5C–XynA is a synthetic, thermally stable, bifunctional enzyme derived from *Thermotoga maritima* and engineered through gene fusion. When xynA was positioned downstream of cel5C, the resulting fusion protein exhibited both cellulase and xylanase activities. However, this activity was absent when xynA was located upstream of cel5C. Typically, the breakdown of complex polysaccharides, such as those in plant cell walls, involves the coordinated efforts of a group of enzymes [70]. To obtain high catalytic activity, a new bifunctional enzyme with mannanase and xylanase activities was engineered. The activity and stability of the developed enzyme with a dual-enzyme ring conformation were determined under adverse conditions, and high catalytic performance was obtained under alkaline pH, extreme temperatures, and freeze-thaw treatment [71]. Gene fusion has produced another artificial bifunctional enzyme with xylanase and mannanase activities. The synergistic action of xylanase and mannanase was observed on hemicelluloses, and the fused enzyme effectively hydrolyzed Luffa cylindrica fibers [72]. The β-xylosidase/exoglucanase enzyme was fused to the carbohydrate-binding modules. After biochemical characterization of the bifunctional enzyme, it was subjected to the degradation of lignocellulosic substrates, including rice and wheat straw, and displayed a high capability for the hydrolysis of a broad spectrum of substrates [73]. Adlakha et al. constructed a bifunctional cellulase/xylanase by fusing the encoding genes isolated from a *Paenibacillus* strain. The enzyme showed secondary structural properties closest to those of native enzymes and could hydrolyze plant biomass [74]. To improve lignocellulose degradation, a gene fusion of endoglucanase from *Teleogryllus emma* and xylanase from *Thermomyces lanuginosus* was designed. The resultant enzyme saccharified pretreated rice straw with a high yield of fermentable sugars, indicating its potential for the degradation of renewable agro-residues and biofuel production [75]. In another report, successful depolymerization of lignocellulosic biomass was reported using a bifunctional enzyme with β-1,3–1,4-glucanase/laccase activities constructed by end-to-end fusion. Synergistic interactions between the two functional domains of the chimeric enzyme became evident when it was applied to break down complex natural substrates [76]. Moreover, a GH7 bifunctional endoglucanase/xylanase was fused and subjected to continuous enzymatic saccharification of sodium carboxymethyl cellulose to produce cello-oligosaccharides and xylo-oligosaccharides [77]. In a previous study, one endoglucanase and one xylanase enzyme were identified from *Gloeophyllum trabeum* and subjected to fusion protein design using a glycine–serine linker. The constructed bifunctional enzyme could degrade popping-pretreated rice straw, corn stover, kenaf, and oak, showing its potential for lignocellulosic biomass hydrolysis and fermentable sugar production [27]. Similarly, a chimeric protein was constructed via end-to-end fusion, which demonstrated endoglucanase and endoxylanase activity. The resulting fusion enzyme demonstrated high thermostability and catalytic activity, making it a good candidate for biorefineries based on plant waste [78]. In addition, rBhcell-xyl was reported as a bifunctional recombinant cellulase/xylanase encoded by *Bacillus halodurans* TSLV1 and created using gene fusion technology. Agro-residues (corn cobs, wheat bran, sugarcane bagasse, sunflower stalks and wheat straw) were tested as substrates for rBhcell-xy to test the level of saccharification [79]. In a recent study, a novel bifunctional xylanase/feruloyl esterase was constructed, which presented enhanced cellulase hydrolysis against different agricultural residues and showed synergistic effects bagasse [80]. As reported in a previous study, bifunctional swollenin and xylanase from *Trichoderma reesei* were fused by connecting the C-terminus of swollenin and N-terminal of xylanase. It was utilized for the hydrolysis of alkali-treated corn cob and demonstrated a much higher yield of reducing sugars compared to the monofunctional enzyme [81].

**Table 2:** List of fused bifunctional enzymes used for lignocellulose-based purposes.

Fused enzyme	Source of genes	Application in lignocellulos industry	Performance metrics or benefits	Ref
Exo/Endo cellulase	*C. saccharolyticus*	Hydrolysis of cellulosic substrates	Specific activity on MCC: 0.167 U/mg (2.2–14× higher), 45–68 % residual activity at 85 °C/2 h, +2.4× activity with Mn²⁺, pH stability (3.5–8.0)	[26]
Cellulase/Xylanase	*Thermotoga maritima*	Hydrolysis of cellulosic substrates	Optimal activity at pH 5.0 and 80 °C, with both enzymatic functions retained	[70]
Mannanase/Xylanase	*Bacillus subtilis*	Production of functional oligosaccharides	Improved stability and synergistic substrate deconstruction efficiency	[71]
1,4-β-xylanase/endo-1,4-β-mannanase	*Pichia pastoris*	Hydrolysis of luffa cylindrica fiber	Enhanced synergistic hydrolysis of hemicellulose from luffa cylindrica fiber	[72]
β-xylosidase/Exoglucanase	*Caldicellulosiruptor saccharolyticus*	Hydrolysis of rice and wheat straw	enhanced stability and saccharification efficiency on diverse lignocellulosic substrates under acidic and high-temperature conditions	[73]
Cellulase/Xylanase	*Paenibacillus*	Hydrolysis of plant-biomass substrates	enhanced activity and broad substrate specificity for plant biomass hydrolysis	[74]
Endoglucanase/Xylanase	Endoglucanase from *Teleogryllus emma*/Xylanase from *Thermomyces lanuginosus*	Saccharification of pretreated rice straw	Specific activities of 306.8 U/mg (CMCase) and 1227.3 U/mg (xylanase), enhancing fermentable sugar yield by 10–20 %, and potential in the biofuel and chemical industries	[75]
β-1,3–1,4-glucanase/laccase	*Bacillus subtilis*	Saccharification of sugarcane bagasse	Bifunctional cellulase/xylanase with 30 % higher laccase activity, enhanced hydrolysis of plant biomass	[76]
Endoglucanase/Xylanase	*Chaetomium thermophilum*	Saccharification of sodium carboxymethyl cellulose and xylan	Bifunctional endoglucanase/xylanase (CTendo7) with 1.91 IU/mg (CMC) and 3.05 IU/mg (xylan) activity, good thermostability, Mn²⁺-enhanced activity	[77]
Cellulase/Xylanase	*Gloeophyllum trabeum*	Hydrolysis of popping-pretreated rice straw, corn stover, kenaf, and oak	Fusion cellulases (Endo5–2CBM-Exo5) with 2.2–14× higher activity on microcrystalline cellulose, enhanced thermostability and pH stability, Mn²⁺-enhanced activity	[27]
Endoglucanase/Endoxylanase	*Fervidobacterium gondwanense*	Hydrolysis plant waste materials	Enhanced activity on glucan and xylan; active from 40 to 100 °C, with improved performance for xylan hydrolysis	[78]
Cellulase/Xylanase	*Bacillus halodurans TSLV1*	Hydrolysis of corn cobs and wheat bran	Thermostable bifunctional enzyme with 2272 U/L CMCase and 910 U/L xylanase, optimal at 60 °C and pH 6.0, high catalytic efficiency (657 mL/mg/min xylanase and 171 mL/mg/min CMCase)	[79]
Xylanase/Feruloyl esterase	*Prevotella ruminicola*	Hydrolysis of wheat straw and sugarcane bagasse	Enhanced lignocellulose hydrolysis, 65 % more sugar production with 40 % cellulase replacement, increased xylan and glucan conversion by 125.1 % and 54.3 %	[80]
Swollenin/Xylanase	*Trichoderma reesei*	Hydrolysis of corn cob	Reduced sugar yield by 42 % on alkali-treated corn cob, 58 % increase with EGII co-treatment, enhance lignocellulose degradation efficiency	[81]
Cellulase/Xylanase/β-glucosidase	*Clostridium thermocellum*	Hydrolysis of alkaline-treated rice straw	Improved cellulose degradation, enhanced glucose production from CMC, RAC, and alkali-treated rice straw, reducing cellobiose accumulation	[82]
Beta-glucosidase gene (BglA)/ endoglucanase (Cel5L)	*(BglA) from Thermotoga maritima /* *Cel5L from Clsotridium thermocellum*	Hydrolysis of carboxymethyl cellulose	Fusion enzyme Cel5L-BglA with 2.0× increased cellulase and 1.8× increased β-glucosidase activity, stable at 80 °C for 2 h, efficient glucose production	[83]
Xylanase/Glucanase/ Feruloyl esterase	*Bacillus aerius strain KW1,*	Bioconversion of corn stover	Fused enzyme with 1.48- to 8.90-fold higher catalytic efficiency, superior to mixed enzymes and commercial cellulase, effective for fermentable sugar production	[84]
XynAm1/ Carbohydrate-binding module 9–2	xynAm1 *from A. niger* and CBM9–2 from *T. maritima*	Synergistic degradation of wheat bran	Fusion enzyme C-F2-X (CBM9–2 + xynAm1) showed 15.91× increased activity, 5.42× higher xylose production from sugarcane xylan, enhanced lignocellulose degradation with cellulase synergy	[85]
Endoxylanase/ Arabinofuranosidase	endoxylanase of *Papiliotrema flavescens* and Arabinofuranosidase of *Aspergillus fumigatus*	Agro-industrial wastes degradation	Enhanced thermostability (10 °C increase), 25 % higher XOS yield, efficient xylo-oligosaccharide production, enhanced by calcium and manganese	[86]
Cellulase Cel5A/ Xylanase Xyn10B,	*Thermotoga maritima.*	Degradation of plant biomass polysaccharides	Cel5A-Xyn10B fusion enzyme showed 2× increased xylanase activity, with 1483 U/mg cellulase activity, synergistic sugar release, stable at 50–90 °C and pH 4.0–9.0; 80 % activity	[87]

Moreover, several enzymes with multiple functions have been synthesized for engineering multifunctional enzymes for biomass industries. In a study by Chen et al. to achieve effective hydrolysis of plant cell walls, a multifunctional cellulase/xylanase/β-glucosidase enzyme was constructed by fusing a bifunctional cellulase/xylanase with a β-glucosidase from *Clostridium cellulovorans*. The fused enzyme was used for in vitro enzymatic hydrolysis of alkali-treated rice straw and generated glucose as a product [82].

### 2.3 Promiscuous enzymes

Promiscuity refers to an enzyme’s capacity to facilitate chemical reactions beyond its primary function [88]. This additional enzymatic role is often termed promiscuous function, underground metabolism[89], or, occasionally, cross-reactivity [90]. The degree of promiscuity describes an enzyme’s specificity level, indicating the extent and diversity of its underground metabolic activity [91]. A straightforward and objective method to determine an enzyme's degree of promiscuity involves analyzing variations in its Enzyme Commission (EC) numbers [92]. The concept of enzyme promiscuity has gained traction, particularly in discussions of enzyme evolution, protein engineering, and biocatalysis [93]. Beyond its evolutionary significance, promiscuity can be purposefully harnessed in the laboratory to develop “novel” enzymatic activities. Practical routes include (i) expanding substrate scope/specificity (e.g., accepting bulkier or chemically distinct substrates)[94], (ii) inverting or improving enantioselectivity and other selectivity parameters (chemo-/regioselectivity)[95], (iii) introducing entirely new catalytic functions through design or directed evolution—illustratively, de novo Diels–Alderases, Kemp eliminases, and retro-aldolases—[96] and (iv) enhancing robustness for non-natural process conditions, such as tolerance to organic solvents, extremes of pH/temperature, or high substrate/product loads[97]. Together, these strategies turn latent side activities into starting points for engineering catalysts tailored to biocatalysis and biomass valorization, and we highlight representative examples in the literature supporting each of these outcomes [93].

Promiscuous enzyme activities are crucial, as they offer a pool of latent catalytic functions that can be utilized when environmental conditions shift and new biochemical capabilities are needed. Through gene duplication followed by divergence under continued selective pressure, these promiscuous functions can evolve into highly specific and efficient enzymes. Even though such activities may not be advantageous at present, they represent potential catalytic tools for future adaptation, although it is inherently unpredictable which functions will become beneficial through evolutionary processes [16]. Enzyme promiscuity plays a pivotal role in the evolution of enzyme superfamilies, transcription factors, and receptor. Under changing environmental conditions, such as the presence of toxins or novel carbon sources, latent promiscuous activities can become biologically significant. Mutations may enhance molecular interactions or amplify their effects, whereas changes in transporter genes may increase the intracellular concentrations of small molecules, promoting interactions with promiscuous proteins. Likewise, mutations in the proteins themselves may improve binding to new targets, such as small molecules or macromolecules. Gene duplication allows one copy of a gene to evolve into a new specialized function, fostering innovation [98,99]. Promiscuous activities have also served as a foundation for creating new biocatalysts, especially for reactions that are otherwise difficult or harmful to achieve using traditional chemical methods [100]. Although promiscuity was largely overlooked 20–30 years ago, when protein research focused on defining specific physiological functions and structure-function relationships, its importance in evolutionary innovation and biotechnology is now widely recognized.

Promiscuous functions are now acknowledged for their significance in both evolutionary biology and medicine and have facilitated numerous biotechnological innovations, even in cases where the functions are physiologically minor or inefficient [100].

Enzyme promiscuity can take several forms, including conditional, substrate, and catalytic promiscuity [101]. Conditional promiscuity occurs when the catalytic behavior of an enzyme is influenced by environmental factors, leading to altered activity under specific conditions. This type of promiscuity can be triggered by a range of parameters, such as extreme temperatures, unusual pH levels, the presence of organic solvents, post-translational modifications, or the scarcity of the enzyme’s natural substrate [102]. For instance, lipases have been extensively used in organic solvents, where both substrate and conditional promiscuity are involved, resulting in a variety of industrial applications. Solid-gas bioreactors provide another example of conditional promiscuity, where the enzyme is immobilized in a dry form on a solid support, and both substrates and products are confined to the gas phase, with no liquid phase involved. This system allows for the precise adjustment of the thermodynamic properties of substrates and water, making it an intriguing approach from a theoretical perspective [101].

Enzymes typically recognize native substrates based on the shapes of their active sites. However, in cases of substrate promiscuity, the conformational flexibility of both the active site and substrate allows the enzyme to bind to non-native substrates. Substrate promiscuity refers to the ability of an enzyme to interact with multiple substrates, reflecting its broad substrate specificity [102]. Examples of substrate promiscuity include coenzyme and lipase substrate promiscuity [101]. In living cells, it is essential to maintain the NADPH—NADP^+^ pool in a reduced state, while the NADH—NAD^+^ pool must be oxidized. Therefore, dehydrogenases must be capable of distinguishing between two cofactor pairs. In addition, lipases are promiscuous in terms of the substrates they accept: they are extensively used to dissolve racemic acids and alcohols, although they do not accept simple alcohols or salts, and they have low enantioselectivity towards chiral acids [103].

In terms of catalytic promiscuity, an enzyme's active site can catalyze multiple reactions. The resulting transformations will vary if bonds are broken or formed in distinct ways or if the transition states for each reaction differ [104]. Dual-activity enzymes demonstrate inherent catalytic promiscuity, as they maintain their original function while acquiring new catalytic abilities [105].

Typically, six enzyme classes–oxidoreductases, transferases, hydrolases, lyases, isomerases, and ligases–are capable of modifying target molecules. Hydrolases have attracted significant attention because of their ability to catalyze reactions previously considered unconventional, including carbon–carbon bond formation, carbon–heteroatom bond creation, oxidative reactions, and novel hydrolytic transformations [106]. Conventional hydrolase-catalyzed reactions, such as hydrolysis, esterification, amidation, and acylation, also demonstrate catalytic promiscuity [101]. The formation of carbon-carbon bonds is a core transformation in organic chemistry. Biocatalysts significantly enhance these reactions, offering distinct advantages over non-enzymatic methods. They enable the synthesis of complex molecules with high chemo-, regio-, and stereoselectivity because multiple stereocenters can be formed under physiological conditions (aqueous environments, neutral pH, and room temperature) [107]. This has become one of the most powerful methods for forming carbon–carbon bonds since the aldol reaction was discovered in 1872. Many promising asymmetric aldol reactions involving hydrolases have recently been reported [[108], [109], [110]].

**Fig. 2:**
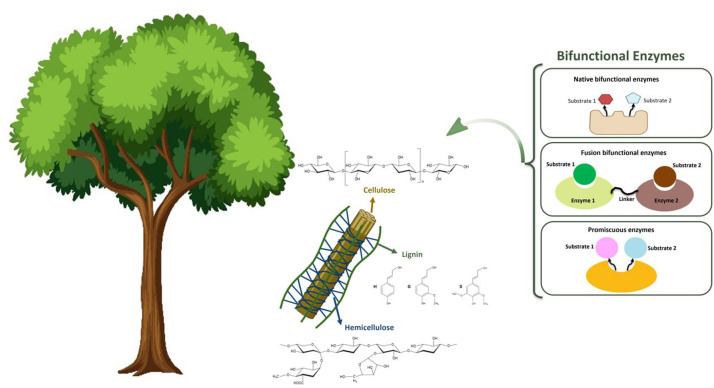
Illustration of lignocellulosic biomass composition (cellulose, hemicellulose, lignin) and the main categories of bifunctional enzymes involved in its degradation. These include native bifunctional enzymes, fusion enzymes composed of two catalytic domains, and promiscuous enzymes with broad substrate specificity. Metagenomic approaches can be used to identify all three types from environmental samples.

Enzymes capable of lignocellulose conversion are prime candidates for directed evolutionary studies because of their substrate promiscuity. Several promising enzymes have been identified to reduce enzyme costs and enhance hydrolytic efficiency in lignocellulosic biomass conversion. In another study, a fungal ligninolytic peroxidase was introduced, which demonstrated promiscuity in the oxidation of a broad range of bulky and recalcitrant lignin substrates. The enzyme was utilized for delignification under extremely acidic pH conditions and was highly stable under these conditions [111]. Min et al. reported the promiscuous activity of tyrosinase, which emphasizes lignin decomposition and the production of value-added products. The promiscuous activity of the enzyme is attributed to the oxidation of veratryl alcohol, a common substrate used for assaying ligninolytic activity and oxidizing dimeric lignin model compounds independently of cation mediators. This enzyme exhibited a high capability for the conversion of biomass and showed a new attitude toward sustainable lignin utilization [112]. Similarly, a phenol hydroxylase from Pseudomonas stutzeri with promiscuous activity has been introduced. Enzyme characterization showed good performance under various pH values and substrate promiscuity. This promiscuous enzyme can convert nine different non-natural substrates, making it attractive for biomass-based applications [113]. To obtain new insights into promiscuous enzymes, Dai et al. reported the production of natural polyhydroxy compounds by hydrolysis of long-chain polyols using glycerol dehydratase and diol dehydratase promiscuous enzymes [114]. In another study, enzymes with dual-functional catalytic domains from a multi-modular glycoside hydrolase were reported to simultaneously target xylan and cellulose. Highlighting its substrate promiscuity, intramolecular synergy was observed in the depolymerization of cellulose in complex lignocellulose corn straw [115]. Moreover, the substrate promiscuity of manganese peroxidases from lignocellulose-degrading fungi was evident when they were subjected to the degradation of four major mycotoxins, which contributed to the large evolutionary diversity of these enzymes. Hence, these new Mn peroxidases have been reported as potential enzymes for the degradation of toxic compounds [116]. In another study, using directed evolution expressed in *Saccharomyces cerevisiae*, thermostable fungal laccases were found to be resistant to high concentrations of cosolvents, demonstrated substrate promiscuity, and could be utilized for potential applications in bioremediation and lignocellulose processing [117]. To highlight the substrate promiscuity of multifunctional enzymes, Liu et al. reported the discovery of an enzyme with amylase, agarase, and carrageenase activities from the marine bacterium *Vibrio alginolyticus*. The enzyme was pH-and thermostable and preferred starch, carrageenan, and agar as substrates. This revealed the evolution of the active sites of enzymes in marine bacteria and proved their reliance on enzymes with the ability to hydrolyze starch [118]. In another study, the calf rumen metagenome was used to discover a multifunctional enzyme from the Family GH43. The enzyme demonstrated β-xylosidase, β-xylanase, and α-L-arabinofuranosidase activities. It has been suggested that the xylose- and arabinose-specific activities of this multifunctional enzyme are due to its evolution and shared promiscuous subsites [119]. Fig. 2 provides a conceptual overview of the major classes of bifunctional enzymes, native, fusion, and promiscuous, alongside their relevance to lignocellulosic biomass deconstruction and metagenome-driven discovery.

## 3 Metagenome-derived bifunctional enzymes for the lignocellulose-based applications

Metagenomics has proven to be an effective approach for exploring gene function and discovering biocatalysts with unique characteristics in diverse environments, including soils, plants, and animals. This technique allows for the direct extraction of DNA from various environments without the need for microbial cultivation [120]. As a culture-independent technique, the richness of metagenomic data makes beneficial screening for enzyme selection. Metagenomics can be divided into functional and sequence-based techniques, respectively. This function-based screening approach involves the extraction and cloning of DNA and its transformation into suitable hosts. Novel genetic elements were detected based on enzyme function. In contrast, the sequence-based screening approach is based on polymerase chain reaction (PCR) and DNA probes of known samples for the detection of desired genes [120]. Additionally, *in silico* screening using computational methods and a multistage pipeline facilitates the analysis of large sets of metagenomic sequences. This approach helps narrow down the candidates, minimize the number of options, and select sequences that align best with the desired performance criteria [37].

**Fig. 3:**
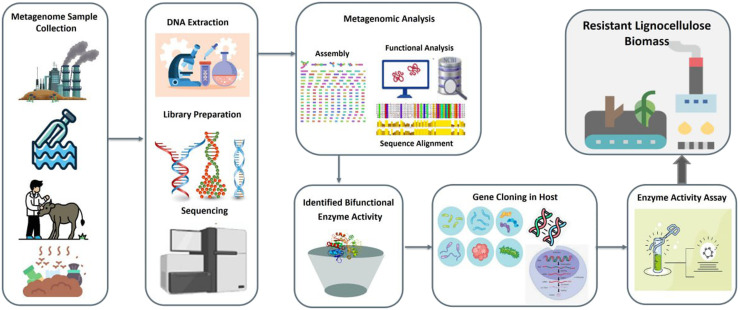
Overview of the metagenomic workflow for identifying bifunctional enzymes. The process includes environmental sampling, DNA extraction, sequencing, functional and sequence-based screening, cloning into suitable hosts, and subsequent enzymatic activity assay on resistant lignocellulosic biomass.

The general steps involved in the metagenomic discovery pipeline for bifunctional enzymes from environmental DNA extraction, sequencing, and bioinformatic analysis to gene cloning, final enzyme activity validation is illustrated in Fig. 3.

**Fig. 4:**
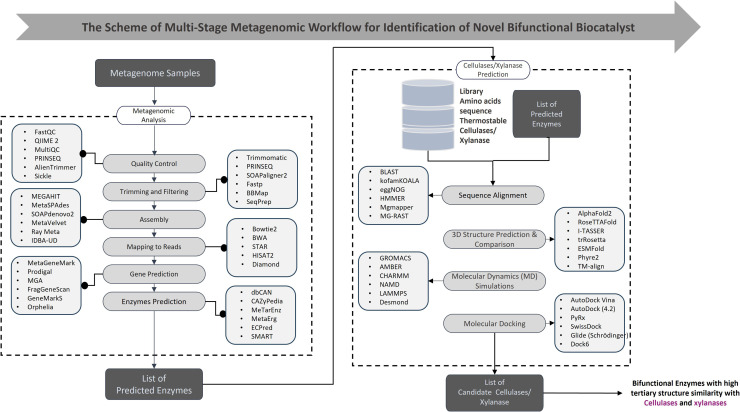
Multi-stage metagenomic workflow for the identification of novel bifunctional enzymes. The pipeline integrates quality control, sequence assembly, gene prediction, functional annotation, 3D structures modeling, molecular dynamics simulations, and docking analysis to prioritize candidate enzymes, such as cellulase/xylanase, with high structural and functional potential.

A schematic representation of the multi-stage metagenomic workflow for the discovery of novel bifunctional enzymes is shown in Fig. 4. The pipeline begins with metagenomic sample acquisition, followed by data quality control, trimming, and assembly. Reads were mapped, genes were predicted, and enzyme candidates were identified using functional annotation tools. Predicted amino acid sequences were subjected to sequence alignment and 3D structural modeling using state-of-the-art prediction algorithms. Molecular dynamics simulations and molecular docking were then applied to evaluate enzyme stability and substrate-binding potential. This process results in a prioritized list of candidate bifunctional enzymes, such as cellulase/xylanase combinations, with high structural similarity to known functional domains [121].

Many studies have focused on the utilization of a metagenomic approach for the discovery of novel bifunctional enzymes, particularly in the rumen microbiota (Table 3). Among the rumen metagenomes, Cel5A and Cel5B were found to be bifunctional. Endoglucanase activity was observed in both enzymes, which made them beneficial in the preliminary stages of degradation of complex polysaccharides at an alkaline pH of 9.0 and a temperature of 65 °C. Both Cel5A and Cel5B are considered non-specific endoglucanases because cellulase and xylanase functions are contained within a single active site, instead of two separate catalytic domains found in classical bifunctional enzymes [122]. Substrate specificity analysis revealed that both enzymes preferred longer oligosaccharides for degradation, indicating that they are endo-cellulases/xylanases. These enzymes exhibit high activity towards xylan and cellulose substrates; therefore, they are suggested for the depolymerization of biomass and biofuel production [122]. Bovine dung (*Chryseobacterium* strain) contains a thermophilic cellulase and auxiliary xylanase activity called CbGH5. This enzyme demonstrated enhanced saccharification effects on straw and spent mushroom substrate. XYN-bw activity was optimal at pH 8, whereas EG-CMC activity was optimal at pH 9. Bioinformatics analysis predicted the presence of two oxidoreductases, a protein-L-isoaspartate O-methyltransferase, and an unknown protein upstream of cbGH5. A cellulolytic strain of *Chryseobacterium* was identified in the dung of cattle fed with cereal straw. When using equivalent enzyme activity units on wheat, rice, corn, and oilseed rape straws, CbGH5 released an additional 3.5–5.03 mg/g of glucose and 1.2–1.83 mg/g of xylose compared to the commercial Novozymes enzyme mixture. CbGH5 can be used for industrial lignocellulosic biomass conversion [123]. Bao et al. identified a bifunctional cellulase with an endo/exo-mode of action. The enzyme can hydrolyze both amorphous (such as carboxymethylcellulose, barley glucan, and lichenan) and crystalline cellulose (filter paper and avicel); thus, it is a promising candidate for the saccharification of biomass. A yak rumen metagenome library was constructed for the identification of β-glucosidase/xylosidase enzymes using a function-based screening approach. The recombinant enzymes were characterized, and their performance in cellulose and hemicellulose saccharification was investigated [124]. A structure–function relationship was observed in a substrate specificity test, and the enzyme generated high amounts of reducing sugars after hydrolysis of cellooligosaccharides [124]. Similarly, the yak rumen metagenome was mined for the discovery of xylanase/endoglucanase, which exhibited synergism with glycosidases in the degradation of xylan and barley glucan .This enzyme successfully degraded pretreated rice straw to produce xylo-oligosaccharides and cello-oligosaccharides, which are necessary for various biomass-based applications such as biofuel production [125]. In another report, a β-glucosidase/xylosidase gene was identified through metagenomic approaches from the yak rumen, and the dual activities were characterized to investigate the potential of the enzyme for the simultaneous saccharification of the lignocellulose substrate and the generation of glucose and xylose [126]. Lee et al. reported the functional characterization of cellulase/hemicellulase enzymes mined from black goat rumen. The ideal reaction conditions and stability profiles of the newly identified bifunctional enzyme were assessed, and its potential use in producing prebiotic konjac glucomannan hydrolysates was explored [35]. A novel bifunctional cellulolytic enzyme was identified by analyzing the metagenome of *Equus burchellii* fecal matter, and the corresponding gene was successfully cloned. The resulting enzyme exhibited broad stability across various temperatures and pH levels, making it suitable for the bioconversion of agricultural residues into valuable products such as single-cell proteins, biofuels, and chemical feedstocks [127]. Nam et al. identified a new bifunctional glucanase–xylanase enzyme through metagenomic analysis, reporting both its sequence and the structural details of its two functional domains. The enzyme architecture closely resembled that of other cellulase family enzymes and demonstrated the ability to hydrolyze both glucan and xylan substrates [128]. Pavarina et al. recently investigated cattle rumen microbiota and identified a novel bifunctional enzyme exhibiting both endo-1,4-β-xylanase and esterase activity. This enzyme was stable within a pH range of 5–6.5, at temperatures from 30 to 45 °C, and was resistant to NaCl and organic solvents [129]. In addition, this novel bifunctional enzyme could degrade xylan and its radical ester, suggesting its potential application as an animal feed additive and for xylooligosaccharide production [129].

**Table 3:** Some novel multi and bifunctional enzymes discovered through metagenomics for application in lignocellulose industries.

Bifunctional enzyme	Metagenomic source	Application in lignocellulos industry	Performance metrics or benefits	Ref
Cellulase/Xylanase	Bovine Rumen	Hydrolysis of cellulose and hemicellulose polymers	Optimal activity at pH 9.0 and 65 °C, effective on long oligosaccharides,	[122]
Cellulase/Xylanase	Dung of cattle fed	Cellulosic biomass conversion	Bifunctional cellulase-xylanase (CbGH5) with 3237 U/mg cellulase and 1793 U/mg xylanase activity, optimal at pH 9, 90 °C, enhanced glucose (3.5–5.03x) and xylose (1.2–1.83x) production from various straws, superior to Novozymes commercial enzymes	[123]
Endo/Exocellulase	Yak rumen	Saccharification of both amorphous and crystalline cellulose	Multifunctional endo/exocellulase Rucel5B with 220 U/mg endoglucanase and 52.9 U/mg exoglucanase activity, efficiently hydrolyzes amorphous and crystalline cellulose, promising for efficient cellulose saccharification	[143]
β-glucosidase/Xylosidase	Yak rumen	Hydrolysis of cellooligosaccharides	The bifunctional enzyme exhibited 218 % and 169 % reduced sugar release in xylan hydrolysis, efficient hydrolysis of cellooligosaccharides, and synergistic activity with xylanase	[124]
Xylanase/Endoglucanase	Yak rumen	Saccharification of pretreated rice straw	Bifunctional enzyme with optimal activities at 65 °C, pH 7.0 (xylanase) and 50 °C, pH 5.0 (endoglucanase), synergistic effects with xylosidase and glucosidase, efficient xylo- and cello-oligosaccharide production	[125]
β-glucosidase/Xylosidase	Yak rumen	Saccharification of cellulose and xylan	50 % reduced sugars; synergistic action with endoxylanase; efficient xylooligosaccharide hydrolysis and cellulose/xylan saccharification	[126]
Cellulase/Hemicellulase	Black Goat rumen	Preparation of prebiotic konjac glucomannan hydrolysates	Effective for prebiotic production, stable under optimal conditions	[35]
Exo/Endo cellulase	*Equus burchelli* Fecal	Bioconversion of agricultural waste materials	Bifunctional exo-1,4-β-glucanase (avicelase) with 6.8 U/mg activity on avicel, optimal at pH 7.0, 35 °C, efficient hydrolysis of avicel and CMC	[127]
Glucanase/Xylanase	Metagenome Library	Hydrolyze both glucan and xylan	Bifunctional CelM2 enzyme with efficient hydrolysis of barley glucan and xylan, promising for industrial biomass degradation and fuel production	[128]
1,4‑β‑xylanase/Esterase	Cattle rumen	Xylooligosaccharide production	Bifunctional endo-1,4-β-xylanase/esterase with 30.96 μmol/min/mg Vmax, stable at pH 5–6.5, 30–45 °C, tolerant to NaCl and organic solvents	[129]
Cellulase/xylanase	Cow rumen	Hydrolysis of the cellulosic and hemicellulosic regions of feedstocks	Stable bifunctional cellulase/xylanase with optimal activity at pH 5, 50 °C, effective degradation of cellulose and hemicellulose, stable under harsh conditions	[37]
Mannanase/Xylanase	Sheep rumen	Hydrolysis various lignocellulosic wastes	Immobilized on magnetic CNCs for improved thermal stability and reusability, 22.2–35.1 % more reducing sugars from lignocellulosic wastes	[139]
Xylanase/Feruloyl esterase	Metagenomic DNA of EMSD5	Hydrolysis various lignocellulosic wastes	High FA (7.31 mg/g) and XOSs (3.21 mg/g) production from agricultural residues, cost-effective	[140]
β -xylosidase/α-l-Arabinofuranosidase	Gut microbiota	Saccharification of lignocellulose	Optimal activity at pH 6.5, 50 °C, salt (3.5 M NaCl) and xylose tolerance, potential for lignocellulose saccharification and industrial applications	[141]
Endocellulase/Exocellulase/Xylanase	Rumen metagenome	Hydrolysis various lignocellulosic substrates	High activity on CMC, birchwood xylan, and oat spelt xylan; optimal at pH 5.0, 37 °C	[142]
Cellulase-Xylanase	Buffalo rumen metagenome	Hydrolysis of agricultural residues	Bifunctional cellulase/xylanase CelXyn2 with optimal activity at pH 6.0, 45 °C, 90.6 % and 86.4 % activity retention at 55 °C	[144]
Glucuronoxylanase/ xylanase	Metagenome of lignocellulolytic bacterial consortium	Lignocellulose saccharification	Specific binding for xylan and arabinose substitutions, 83 % reduction in kcat/Km with CrCBM2 truncation, 5x improvement in hydrolysis efficiency	[145]
xylosidase/arabinosidase	Soil metagenome	Biomass waste degradartion	Bifunctional xylosidase/arabinosidase with 60 °C optimal activity; efficient xylose (1.35 mg) and arabinose (2.24 mg) production from maize biomass; promising for bio-manufacturing	[146]

To effectively handle large-scale datasets, metagenomic analyses must be cost-efficient and time-saving in terms of laboratory work requirements. In this context, advanced sequencing technologies combined with bioinformatics play a crucial role in identifying bifunctional enzymes owing to their enhanced capability to filter and analyze sequencing data. Furthermore, ongoing advances in biotechnology have led to the emergence of innovative tools for enzyme discovery using metagenomic data analysis. By leveraging machine learning regression models and computational strategies, these tools enable the systematic exploration of microbial diversity, helping to identify high-performance enzymes optimized for industrial applications. These prediction tools can reduce the time and cost of discovering enzymes with desired properties by highlighting the targeted screening of high-throughput data [[130], [131], [132]]. To date, several metagenome-derived enzymes applicable to lignocellulose-based industries have been identified using this strategy. For instance, a multi-step *in silico* screening approach, such as homology modeling, molecular docking, and stability prediction, was utilized to identify endo-β−1,4-glucanase enzymes with high stability under extreme temperature and pH conditions for use in the bioconversion of lignocellulosic biomass under harsh conditions [133]. Furthermore, a novel glucose-tolerant β-glucosidase was identified through computationally guided experiments for the bioconversion of lignocellulosic biomass [134]. Furthermore, metagenomic data of the rumen were mined for the *in silico* screening of two thermostable xylanases with high catalytic activity under mild conditions and the ability to liberate fermentable sugars from biomass [135,136]. Similarly, cellulases were successfully identified from the metagenome using a potential in-silico screening pipeline and bioinformatic approach to be utilized in biorefineries and lignocellulose bioconversion-based technologies [137,138]. Using this pipeline, various bifunctional enzymes have been mined from metagenomic data. A newly discovered bifunctional mannanase/xylanase biocatalyst exhibited excellent hydrolytic efficiency on corn cobs and was capable of breaking down sugar beet pulp, coffee residue, and rice straw, yielding high concentrations of reducing sugars [139]. In another study, a novel and stable bifunctional cellulase/xylanase was identified through *in silico* screening of rumen metagenomic sequencing data and was applied for the bioconversion of lignocellulosic biomass [37]. To enhance the production of ferulic acid and xylo-oligosaccharides, a bifunctional xylanase/feruloyl esterase was identified. This multi-domain recombinant enzyme has been employed to break down various biomass types, including insoluble wheat arabinoxylan, de-starched wheat bran, ultrafinely ground material, corn stover, and steam-exploded corncobs [140]. Xu et al. described the development of a bifunctional β-xylosidase/α-L-arabinofuranosidase belonging to the GH43 family, which demonstrated stability at high salt and xylose concentrations, as well as under alkaline pH conditions [141]. Ruminal bacteria were selected as the source of the metagenome for the identification of multifunctional enzymes. A robotic high-throughput screening system was used to screen for a multifunctional enzyme with endocellulase/exocellulase/xylanase activities. In addition, the enzyme exhibited a wide range of substrate spectra, and its performance was stimulated in the presence of different metal ions. These properties make this enzyme a promising candidate for improving the degradation of cellulosic biomass in biorefineries [142].

## 4 Conclusion and future prospects

The efficient breakdown of polysaccharide components in lignocellulosic biomass into fermentable sugars remains a major bottleneck in biomass-based industry. Overcoming this challenge requires the synergistic action of multiple enzymes, highlighting the growing importance of discovering and characterizing bi- and multifunctional enzymes. In this regard, the identification of such enzymes from diverse sources, including metagenomic libraries, and the construction of fusion enzymes through protein engineering represent promising strategies for advancing our understanding of these biocatalysts and their pivotal roles in biomass valorization. Bifunctional enzymes can be broadly classified into native, metagenome-derived, and promiscuous enzymes based on their structural origin and catalytic function. Their multifunctional nature not only enhances substrate specificity but also contributes to operational simplicity and cost efficiency in enzymatic hydrolysis processes. In the near future, bifunctional and multifunctional enzymes are expected to play an increasingly significant role in agro-industrial applications and biofuel production because of their potential to streamline biomass conversion. A comprehensive understanding of dual-function enzymes, particularly their catalytic mechanisms, structural domains, and synergistic activities, can substantially facilitate their rational design and targeted discovery from natural environments. The identification of enzymes with enhanced hydrolytic capacity, operational stability across a wide pH and temperature range, and resistance to extreme industrial conditions can significantly improve the biodegradation efficiency of lignocellulosic biorefineries. Despite their potential, research on bifunctional enzymes remains limited, emphasizing the urgent need to expand the investigation in this emerging field. Future studies focusing on structure-function relationships, bioinformatics-guided screening, and synthetic biology approaches will be essential for harnessing the full potential of these enzymes in sustainable bioprocessing.

## CRediT authorship contribution statement

**Razieh Goudarzi:** Writing – original draft, Methodology, Investigation. **Donya Afshar Jahanshahi:** Writing – original draft, Methodology, Investigation. **Arashk Kavousi:** Visualization, Investigation. **Shohreh Ariaeenejad:** Methodology, Conceptualization.

## Declaration of competing interest

The authors declare that they have no known competing financial interests or personal relationships that could have appeared to influence the work reported in this paper.

## Data Availability

No data was used for the research described in the article.
